# Combinatorial effects of tryptophan derivatives serotonin and indole on virulence modulation of enteric pathogens

**DOI:** 10.1128/mbio.02067-25

**Published:** 2025-08-25

**Authors:** Mehmet Ali Hoskan, Vanessa Sperandio

**Affiliations:** 1 Department of Medical Microbiology and Immunology, University of Wisconsin – Madison 732057, Madison, Wisconsin, USA; 2 Department of Microbiology, University of Texas Southwestern Medical Center 12334, Dallas, Texas, USA; Georgia Institute of Technology, Atlanta, Georgia, USA

**Keywords:** enterohemorrhagic *E. coli *(EHEC), *Citrobacter rodentium*, serotonin, indole, locus of enterocyte effacement (LEE)

## Abstract

There is a wealth of signals present in the human gut that mediate host-microbiota communication and intersect with the gut-brain-axis. There is differential spatial localization of the tryptophan derivatives serotonin and indole in the gut, which are important cues for enteric pathogens to find their colonization niche. Both signals are sensed by enteric pathogens such as enterohemorrhagic *Escherichia coli* (EHEC) and *Citrobacter rodentium*, a murine pathogen extensively employed as a surrogate animal model for EHEC. EHEC and *C. rodentium* virulence determinants include genes necessary for the attaching and effacing (AE) lesion formation on enterocytes, which are contained within the locus of enterocyte effacement (LEE) pathogenicity island. Both signals inhibit LEE expression *in vitro* and during mammalian infection. The roles of indole and serotonin in virulence have been initially interrogated separately. Here, we show the combinatorial effect of these signals antagonizing each other’s activity on bacterial virulence *in vitro* and during murine infection. The role of both signals *in vivo* was interrogated by manipulating the serotonin levels in mice through pharmacological and genetic strategies to increase the levels of serotonin in the gut by inhibiting the serotonin reuptake transporter (SERT). Simultaneously, indole levels were altered through infection with *C. rodentium* strains that either produce or don’t indole or recolonizing the microbiota with WT or Δ*tnaA* (does not produce indole) strains of *Bacteroides thetaiotaomicron*. Individually increasing the levels of serotonin and indole in the gut decreased *C. rodentium* pathogenesis. However, when both signals were elevated simultaneously, they antagonized each other’s activity.

**IMPORTANCE:**

Pathogens sense a plethora of signals within the gut to successfully establish colonization by precise regulation of virulence gene expression within the right niche. Our study shows that it is crucial to not disregard the interaction of different signaling mechanisms to understand the complexity of virulence regulation in enteric pathogens. Even though serotonin and indole are both tryptophan derivatives with similar structures that individually decrease bacterial virulence, combinatorial sensing of these two signals cancels out each other’s effect. Understanding these sensing mechanisms provides a better insight into potential therapeutic approaches against enteric infections.

## INTRODUCTION

Enteric pathogens gage the fluctuations in the availability of chemical signaling molecules, nutrients, and oxygen in the gut to modulate the expression of their virulence genes toward successful host colonization (1). Enterohemorrhagic *Escherichia coli* (EHEC) serotype O157:H7 is a human enteric pathogen. EHEC is a highly efficient pathogen with a very low infectious dose of 10–100 colony-forming units (CFUs) (2, 3). It employs a type three secretion system (T3SS), a needle-like structure that translocates bacterial effectors within the host cell. The concerted action of these effectors leads to effacement of the microvilli, and actin rearrangement in epithelial cells that form pedestal-like structures promoting bacterial adhesion. These structures are named attaching and effacing (AE) lesions (4). The T3SS is encoded by the locus of enterocyte effacement (LEE) pathogenicity island. The LEE consists of 41 genes, most of them clustered into 5 operons (*LEE1* to *LEE5*) (5). The *ler* gene within the *LEE1* operon encodes the master regulator of the LEE genes (6). Regulation of *ler* transcription is controlled by a plethora of signals and small molecules present in the GI environment. These signals are derived from the host and the microbiota and are sensed and integrated to modulate the precise expression of the T3SS within the right niche; thus, they provide a survival advantage to EHEC when competing with other residents of the microbiota (1, 7). EHEC also produces Shiga toxin (Stx) that is responsible for hemolytic uremic syndrome (HUS) in patients (2, 3).

In the gastrointestinal tract (GI), there are different tryptophan derivatives that serve as signaling molecules for the host and the microbiota (8). Serotonin, also known as 5-hydroxytryptamine (5-HT), is a tryptophan derivative neurotransmitter produced by the tryptophan hydroxylase (TpH1) enzyme in enterochromaffin cells. Enterocytes express the serotonin reuptake transporter (SERT) that removes serotonin from the intestinal mucosa. 95% of the serotonin in the human body is synthesized in the gut (9). Perpetual production of serotonin by enterochromaffin cells and its constant reuptake by enterocytes through SERT lead to homeostasis of serotonin levels in the gut. Serotonin levels are estimated to be around 5 µM in the lumen and 30 µM in the epithelial tissue. The abundance of serotonin in the GI environment makes it an important signal for enteric pathogens (10).

Indole is another tryptophan derivative produced by members of the microbiota (11). The tryptophanase enzyme synthesizes indole from tryptophan. Tryptophanase is encoded by the *tna* operon and different members of microbiota such as *Escherichia coli* and *Bacteroides thetaiotaomicron* harbor the *tna* operon to produce indole (11, 12). Indole levels in human stool range between 250 and 1000 µM, and in liquid culture, *E. coli* produces 500 µM of indole (13, 14). The indole concentration in the gut is higher in the lumen and decreases at the epithelial lining. Indole is continuously produced by the microbiota and absorbed by the host cells, which leads to a gradient of indole concentration in the intestine (14).

**Fig 1:**
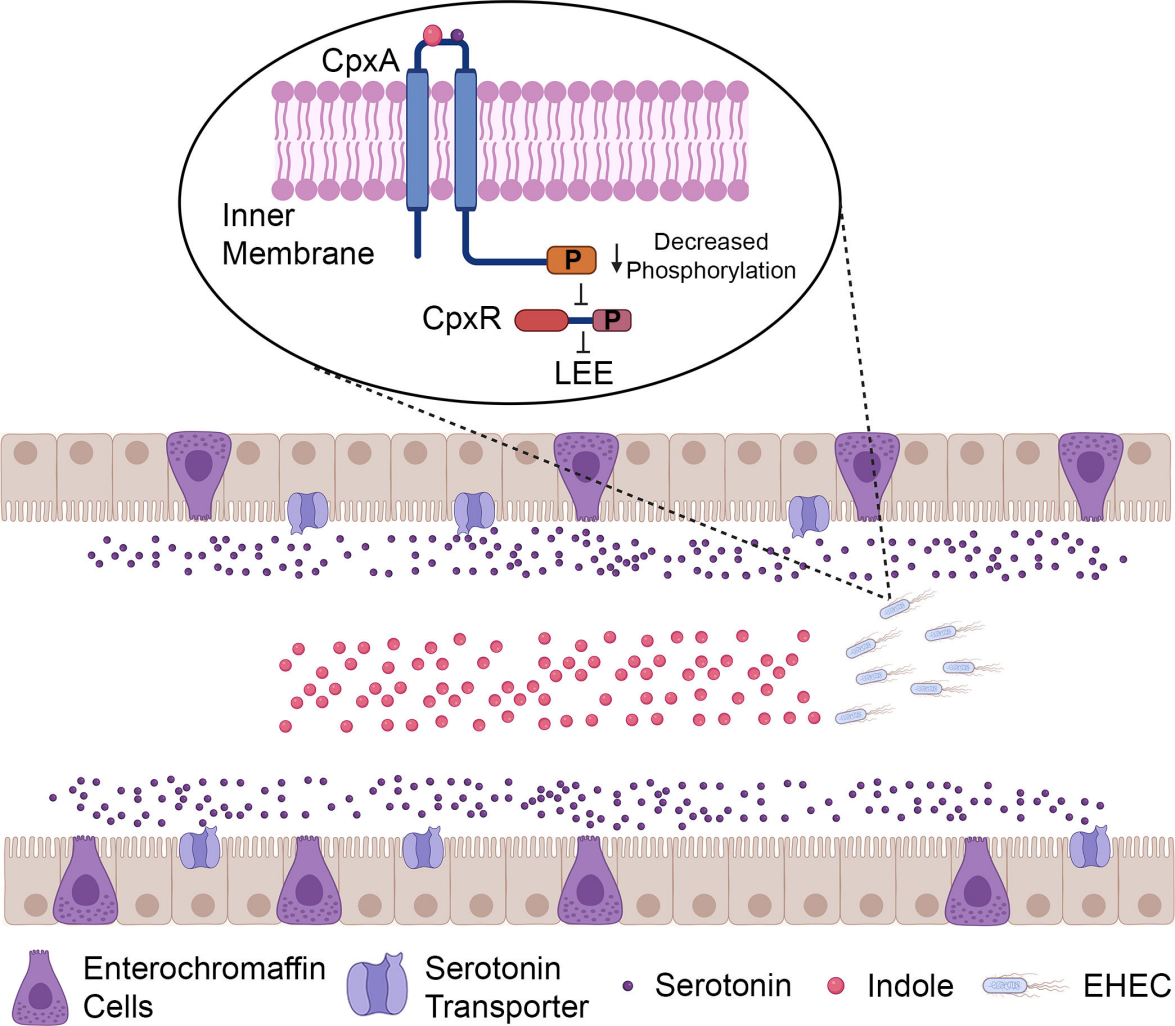
EHEC senses tryptophan derivatives serotonin and indole through the CpxAR two-component system to downregulate its virulence gene expression. Indole is produced and secreted by the members of the microbiota, and indole concentrations are higher in the lumen. Serotonin is produced by enterochromaffin cells in the epithelial layer, and serotonin reuptake transporters on enterocytes uptake serotonin; serotonin levels are higher on the epithelial mucosa. Serotonin and indole decrease the expression of the LEE; sensing of serotonin and indole leads to dephosphorylation of the CpxA histidine kinase and its response regulator CpxR. Dephosphorylated CpxR cannot activate LEE expression. Created in BioRender.

Serotonin and indole are both sensed through the CpxAR two-component system. CpxA is a sensor histidine kinase that senses bacterial envelope stress and phosphorylates CpxR, its cognate response regulator. Upon phosphorylation, CpxR activates the expression of genes encoding proteins important for envelope maintenance and LEE genes. Sensing of serotonin and indole leads to dephosphorylation of CpxA; thus, CpxR gets dephosphorylated, which leads to a decrease in the expression of the LEE genes (Fig. 1) (10, 14).

Here, we show that the inhibitory effects of serotonin and indole on the virulence of the enteric pathogens EHEC and *C. rodentium* are not additive. Even though serotonin and indole are both sensed through the CpxAR two-component system and decrease LEE gene expression and production of T3SS *in vitro* and *in vivo*, their combinatorial effect antagonizes each other. Utilizing SERT knockout animals and pharmacological inhibition of SERT alongside infection with different strains of *C. rodentium* and recolonization of microbiota with different strains of *B. thetaiotaomicron* to manipulate the indole levels, we demonstrated that increasing serotonin and indole levels together cancels out their inhibitory activity on *C. rodentium* virulence and pathogenesis *in vivo*.

## RESULTS

### Serotonin and indole decrease EHEC virulence gene expression individually but antagonize each other

We previously reported that serotonin and indole individually inhibit EHEC virulence (10, 14). Indole is produced by the members of microbiota residing in the lumen of the GI tract. The concentration of indole is significantly higher in the lumen when compared to colonic tissue (14). Notably, 95% of serotonin is produced in the gut by enterochromaffin cells, which leads to higher serotonin concentrations in the epithelial mucosa (9, 10, 15). Given that serotonin and indole are both tryptophan derivatives, with similar chemical structures, and that they are sensed through the CpxAR two-component system in bacterial cells, we hypothesized there could be an interplay between these two signals.

**Fig 2:**
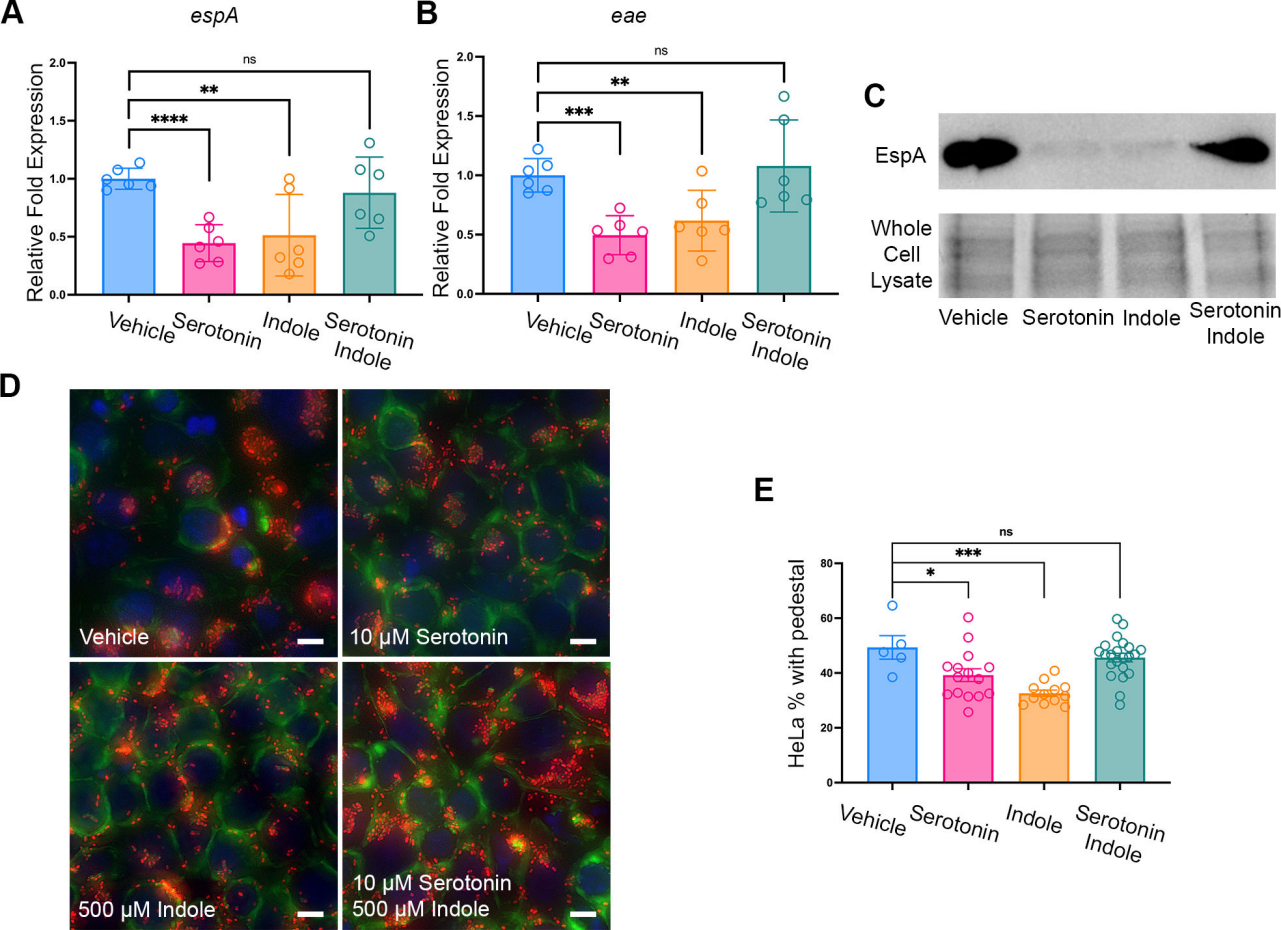
Serotonin and indole decrease EHEC virulence gene expression individually but antagonize each other. (**A, B**) qRT-PCR analysis comparing expression of virulence genes *espA* and *eae* in WT EHEC treated with vehicle, 10 µM serotonin, 500 µM indole, or both (*n* = 3 biological samples, 6 technical replicates). Error bars represent standard deviations (SD). *P*-value was calculated using unpaired *t* test. *P < 0.05; **P < 0.01; ***P < 0.001; ns, not significant. Fold changes were calculated relative to *rpoA* as an internal control. (**C**) Western Blot of EspA of EHEC treated with vehicle, 10 µM serotonin, 500 µM indole, or both (*n* = 3). (**D**) Fluorescein actin staining analysis. HeLa cells were infected with EHEC with a plasmid encoding *mCherry* and treated with vehicle, 10 µM serotonin, 500 µM indole, or both. After 5 h of infection, cells were washed and stained with FITC-phalloidin to visualize actin (green), mounted with Prolong Diamond Antifade with DAPI to visualize nuclei (blue), and bacteria were visualized by detection of mCherry (red). Pedestals were visualized in green. Scale bars, 50 µm. (**E**) Quantitative analysis of the HeLa cells with pedestal formation by EHEC. Each field contains approximately 100 cells. The percentage of HeLa cells with pedestal formation was quantified (*n* = 3 biological samples). *P*-value was calculated using unpaired *t* test. Error bars represent standard deviations (SD). *P < 0.05; **P < 0.01; ***P < 0.001; ns, not significant. Data are representative of at least three biological replicates.

To test whether serotonin and indole have a combinatorial effect, we grew EHEC in the absence or presence of 10 µM serotonin and 500 µM indole individually or together. It’s been reported that the serotonin concentration in the gut is estimated to be around 5–30 µM and *E. coli* is estimated to produce 500 µM of indole when grown *in vitro*, and concentrations of indole in human stool are reported to vary between 250 µM and 1 mM. Hence, these are physiological concentrations of these signals in the gut. EHEC had no growth differences in the absence or presence of these signals in these concentrations (Fig. S1A). Using qRT-PCR to quantify the expression of the LEE virulence genes *espA* and *eae* and Western blots to quantify the production of the EspA protein, we observed a significant decrease in virulence of EHEC when grown with 10 µM serotonin and 500 µM indole. However, when serotonin and indole were supplemented together, we observed no significant change in virulence expression (Fig. 2B through D). Secretion of the LEE-encoded EspB protein using ELISA assays decreased in the presence of serotonin or indole, but there was no decrease when both signals were added together (Fig. S1B). Congruent with the LEE expression data, serotonin or indole individually decreased the formation of AE lesions by EHEC on HeLa cells, while there was not a significant decrease when both signals were together (Fig. 2E and F).

### The serotonin and indole regulons

**Fig 3:**
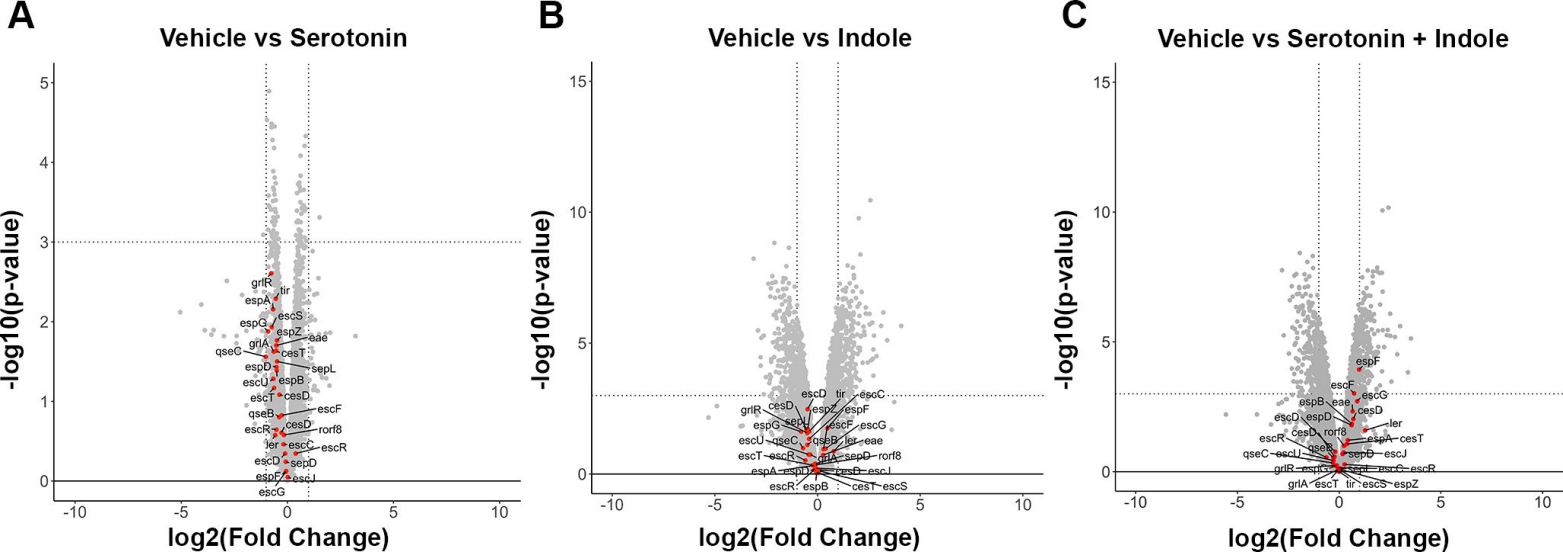
Serotonin and indole regulons. Volcano plot indicating differentially regulated genes when comparing EHEC treated with vehicle to EHEC treated with (**A**) 10 µM serotonin, (**B**) 500 µM indole, or (**C**) both. (**A**) Downregulation of most genes in the locus of enterocyte effacement (LEE) was observed in EHEC treated with 10 µM serotonin (**A**) and 500 µM indole (**B**) but not in 10 µM serotonin and 500 µM indole together (**C**).

To investigate the transcriptome of serotonin and indole, we performed RNASeq in the presence and absence of these signals (GEO accession no. GSE296573). In the presence of serotonin, a total of 531 genes were downregulated, and 481 genes were upregulated; in the presence of indole, 1,310 genes were downregulated, and 1,201 genes were upregulated. In the presence of both, 1,203 genes were downregulated, and 1,123 genes were upregulated. Downregulated genes include the LEE genes in the serotonin and indole treated groups but not when EHEC cells are treated with both (Fig. 3). In addition to LEE encoded genes, *qseB* and *qseC* genes that encode for the two-component system that responds to epinephrine and norepinephrine and autoinducer 3 (AI-3) were also downregulated, suggesting an intersection with sensing mechanisms of other signals (16–18) (Fig. S2).

### Serotonin and indole regulation of *C. rodentium* virulence

*C. rodentium* is a murine pathogen that is commonly used as a surrogate animal model for EHEC infections in mice (19–21). We employed a *C. rodentium* strain engineered to produce Shiga toxin (we refer to this strain as the wild-type, WT), which successfully mimics EHEC infection in mice by forming AE lesions and causing kidney damage and lethality (22). To investigate the physiological effect of combinatorial sensing of serotonin and indole in *C. rodentium*, we first tested virulence expression *in vitro. C. rodentium* was grown for 2 h without the addition of serotonin or indole, and 500 µM indole or vehicle was added into the growth medium. Cells were grown for 2 h more, and then 10 µM serotonin or vehicle was added into the growth medium, and cells were harvested at the 6 h time point when they reached late logarithmic phase. qRT-PCR of *ler*, encoding the master regulator of the LEE pathogenicity island, was decreased with each signal alone, and these signals antagonized each other when combined, as observed with EHEC LEE expression (Fig. S3A). We also observed the same effect on the secretion of the LEE-encoded EspB protein (Fig. S3B).

### Indole production decreases *C. rodentium* pathogenesis in C57BL/6 mice

We previously reported that elevated indole levels in the gut decrease *C. rodentium* virulence in C3H/HeJ mice (14). The C3H/HeJ mouse strain is highly susceptible to *C. rodentium* infection, which makes it a great model organism for *C. rodentium* infection studies (23). C57BL/6 mice are more resistant to infection, but they are commonly used in transgenic mouse studies, which provides genetic tools to manipulate serotonin levels in the gut (24–26). Before manipulating indole levels in parallel to serotonin levels in C57BL/6 mice, we assessed whether the inhibitory effect of indole on *C. rodentium* pathogenesis previously observed in C3H/HeJ mice was reproducible in C57BL/6 mice. Congruent with our previous studies, we observed a decrease of *C. rodentium* pathogenesis in C57BL/6 mice caused by indole production.

**Fig 4:**
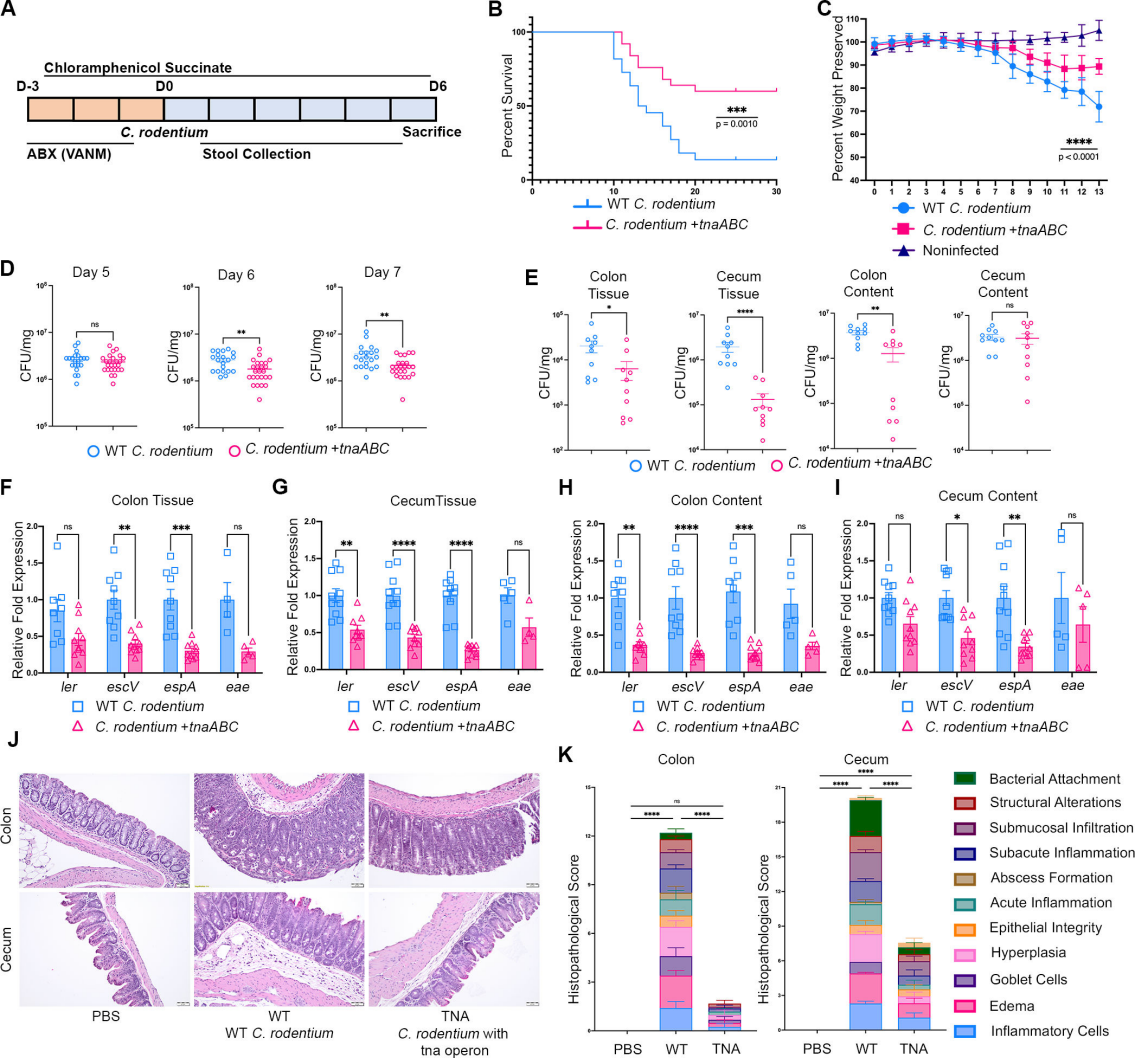
Indole production decreases *C. rodentium* pathogenesis in C57BL/6 mice. (**A**) Schematic of *C. rodentium* murine infection experiments with C57BL/6 mice. D, day. Prior to infection day, animals were treated with a cocktail of vancomycin, ampicillin, neomycin, and metronidazole for three consecutive days. Additionally, animals were given 1 mg/mL chloramphenicol succinate in drinking water flavored with 1% sucrose and grape-flavored Kool-Aid mix throughout the experiment. Animals were infected with either WT *C. rodentium* or *C. rodentium + tnaABC* strain that harbors the *tna* operon to produce indole. (**B**) Survival curves of C57BL/6 mice infected with WT *C. rodentium* (*n* = 22) and *C. rodentium + tnaABC* strain producing indole (*n* = 25). PBS mock-infected animals were used as a negative control (*n* = 5). Statistics are shown using the log-rank (Mantel-Cox) test; *n* = number of mice. (**C**) Weight comparison of C57BL/6 mice infected with WT *C. rodentium* (*n* = 22) and *C. rodentium + tnaABC* strain producing indole (*n* = 25). Statistics are shown using mixed-effect analysis; *n* = number of mice. (**D**) Enumeration of colony-forming units (CFU) of *C. rodentium* loads recovered from stools collected from WT *C. rodentium* infected mice (*n* = 22) and *C. rodentium + tnaABC* infected mice (*n* = 25) on day 5, day 6, and day 7 post-infection. Error bars represent standard errors of the mean (SEM). Groups were compared using unpaired *t*-test. **P < 0.01; ns, not significant. (**E**) Enumeration of CFUs of *C. rodentium* loads recovered from tissue and content samples of the colon and cecum of the mice sacrificed at day 6 post-infection. *n* = 10 mice per group. Error bars represent standard errors of the mean (SEM). Experimental groups were compared using unpaired *t*-test. *P < 0.05; **P < 0.01; ****P < 0.0001; ns, not significant. (**F–I**) qRT-PCR analysis comparing expression of virulence genes encoded in the LEE pathogenicity island in the RNA from colon tissue (**F**), cecum tissue (**G**), colon content (**H**), and cecum content (**I**) of the mice sacrificed at day 6 post-infection. *n* = 5 or 10 mice per group. Error bars represent standard errors of the mean (SEM). *P*-values were calculated using multiple unpaired *t*-tests followed by multiple comparisons using the Bonferroni-Dunn method; *P < 0.05; **P < 0.005; ***P < 0.001; ****P < 0.0001; ns, not significant. Fold changes were calculated relative to *rpoA* as an internal control. (**J**) Colon tissues and cecum tissues of C57BL/6 mice infected with WT *C. rodentium* and *C. rodentium + tnaABC* at day 6 post-infection, stained with hematoxylin and eosin. Scale bars, 50 µm. (**K**) Blinded histopathology scores of C57BL/6 mice infected with WT *C. rodentium* and *C. rodentium + tnaABC*. The PBS mock infected group was scored zero. *n* = 5 mice per group. *P*-value is calculated using two-way ANOVA followed by Bonferroni’s multiple comparison test. ****P < 0.0001.

Although *C. rodentium* is a vastly used as a murine model for EHEC infections, unlike EHEC, it doesn’t encode the tryptophanase enzyme that produces indole. We used a *C. rodentium* strain engineered to produce indole by insertion of the EHEC *tna* operon within the *lacZ* locus (this locus is not necessary for infection [14]) of *C. rodentium*; the indole producing strain is referred to as *C. rodentium + tnaABC* (14). Prior to experimentation, WT and indole producing *C. rodentium* strains were tested for indole production using *p*-Dimethylaminocinnamaldehyde (DMACA) indole reagent droppers and BBL indole reagent droppers (14). To observe the effect of indole produced by *C. rodentium*, microbiota was depleted with antibiotic administration as previously described (14, 27). The *C. rodentium* strains used are resistant to chloramphenicol. To prevent restoration of the microbiota following its depletion, we supplemented chloramphenicol in the drinking water. We inoculated animals with either WT *C. rodentium* or *C. rodentium + tnaABC* (Fig. 4A). Animals infected with WT *C. rodentium* were more susceptible to infection and presented more pronounced weight loss compared to animals infected with *C. rodentium + tnaABC,* which had a higher survival rate and preserved more of their weight throughout the experiment (Fig. 4B and C). Bacterial burden in the stools, as well as in the contents and tissues collected from the colon and cecum of *C. rodentium + tnaABC* infected animals were also significantly lower compared to animals infected with WT *C. rodentium* (does not produce indole) (Fig. 4D and E). Animals infected with *C. rodentium + tnaABC* also presented reduced pathology upon infection (4J and 4K). Congruently, we observed a significant decrease in the expression of the LEE genes in the RNA extracted from tissues and contents from the colon and cecum of the animals infected with *C. rodentium + tnaABC* compared to WT *C. rodentium* (Fig. 4F through I).

### Indole and serotonin antagonize each other’s activity in decreasing *C. rodentium* pathogenesis in C57BL/6 mice

**Fig 5:**
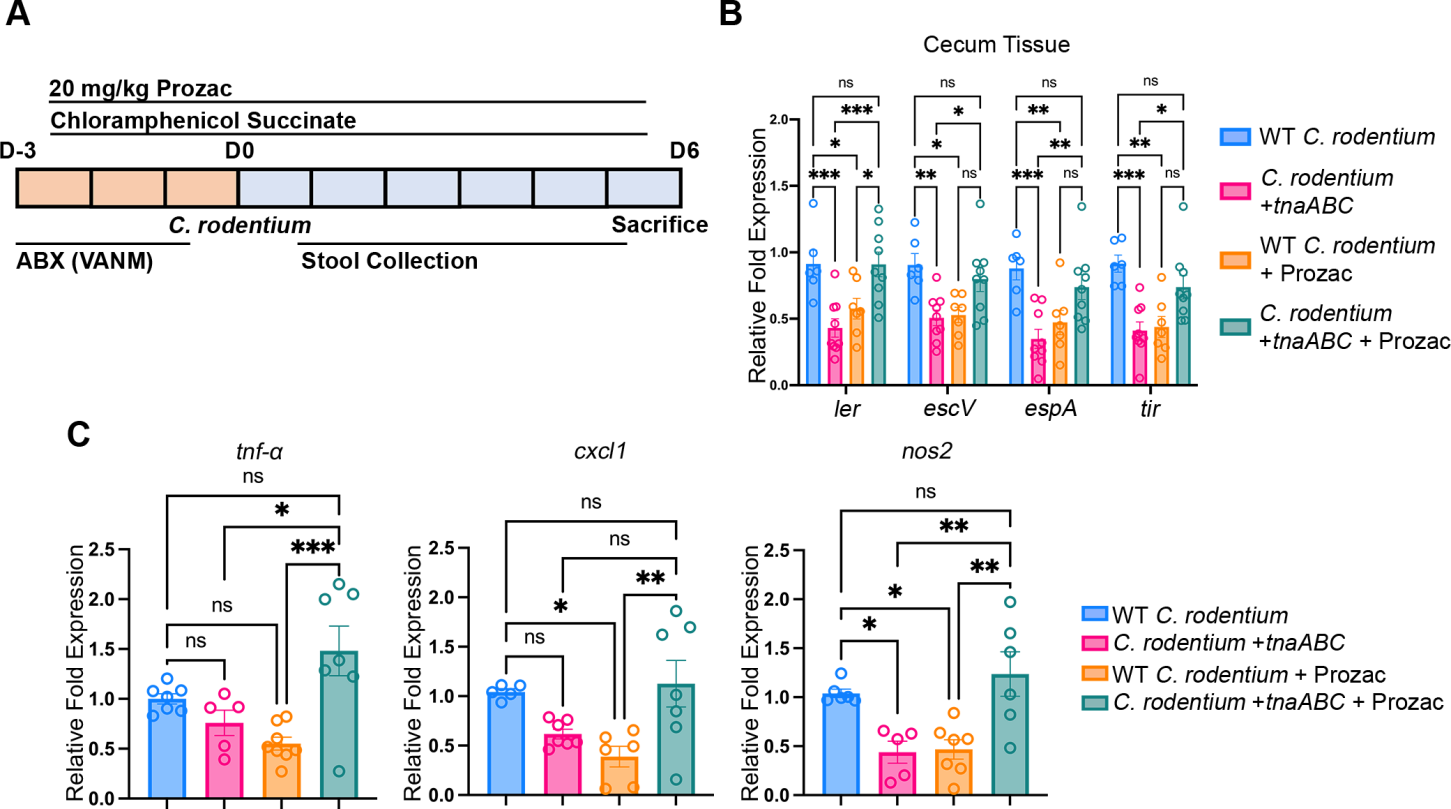
Manipulation of serotonin levels with Prozac prevents the decrease in *C. rodentium* pathogenicity caused by indole production. (**A**) Schematic of *C. rodentium* murine infection experiments with C57BL/6 mice. D, day. Prior to infection, animals were treated with a cocktail of vancomycin, ampicillin, neomycin, and metronidazole for three consecutive days. Additionally, animals were administered 20 mg/kg Prozac through oral gavage and given 1 mg/mL chloramphenicol succinate in drinking water flavored with 1% sucrose and grape-flavored Kool-Aid mix throughout the experiment. Animals were infected with either WT *C. rodentium* or *C. rodentium +tnaABC* strain that harbors the *tna* operon to produce indole. *n* = 8 mice per group. (**B**) qRT-PCR analysis comparing expression of virulence genes encoded in the LEE pathogenicity island in the RNA from cecum tissue collected from mice infected with WT *C. rodentium* and *C. rodentium +tnaABC* that are administered Prozac or PBS as vehicle and sacrificed at day 6 post-infection. Error bars represent standard errors of the mean (SEM). *P*-values were calculated using two-way ANOVA followed by Bonferroni’s multiple comparison test. *P < 0.05; **P < 0.005; ***P < 0.001; ****P < 0.0001; ns, not significant. Fold changes were calculated relative to *rpoA* as an internal control. (**C**) qRT-PCR analysis comparing expression of inflammation markers in the extracted RNA from cecum tissue collected from mice infected with WT *C. rodentium* and *C. rodentium +tnaABC* that are administered Prozac or PBS as vehicle and sacrificed at day 6 post-infection. Error bars represent standard errors of the mean (SEM). *P*-values were calculated using one-way ANOVA followed by Bonferroni’s multiple comparison test. *P < 0.05; **P < 0.005; ***P < 0.001; ns, not significant. Fold changes were calculated relative to *gapdh* as an internal control.

Serotonin is synthesized by enterochromaffin cells, released into the intestinal layer lamina propria, and SERT removes the serotonin from the intestinal mucosa (9). Pharmacological inhibition of SERT leads to accumulation of serotonin in the gut,, thus leading to a decrease in *C. rodentium* pathogenesis (10). To investigate the combinatorial effect of serotonin and indole during murine infection, we administered fluoxetine (Prozac) to C57BL/6 mice infected with either WT *C. rodentium* or *C. rodentium +tnaABC* after microbiota depletion with antibiotics (Fig. 5A). Expression of LEE genes (*ler*, *escV*, *espA*, *tir*) was decreased in the cecum tissues of animals that received Prozac and animals infected with *C. rodentium +tnaABC*. In contrast, when animals infected with *C. rodentium +tnaABC* were administered Prozac, when both indole and serotonin levels were elevated, expression of LEE genes was the same as it was in animals infected with WT *C. rodentium* (Fig. 5B). The same effect was observed in the expression of inflammation markers (*tnf-a*, *cxcl1*, *nos2*) in the host, where we observed a decrease in the *C. rodentium +tnaABC* infected mice and Prozac administered mice but not when both *C. rodentium +tnaABC* infection and Prozac administration were done together (Fig. 5C).

### The absence of microbiota in gnotobiotic C57BL/6 mice interferes with the effect of serotonin and indole on *C. rodentium* pathogenesis

The microbiota produces high concentrations of indole, and its presence also promotes the biosynthesis of serotonin (28). The antibiotic depletion of microbiota is an important tool for eliminating interference of microbiota with serotonin and indole levels, but after antibiotic treatment and infection of animals, even though we try to minimize the recolonization through consistent administration of chloramphenicol in drinking water, mice still get recolonized. We also assessed the combinatorial effect of these two signals using gnotobiotic mice. In gnotobiotic mice, the effect of serotonin and indole occur very early in the infection. CFU loads from the stools showed a significant decrease in animals infected with *C. rodentium +tnaABC* compared to WT *C. rodentium*. Administration of Prozac that enhances the levels of gut serotonin by inhibiting SERT also led to decreased loads from the stool of animals infected with WT *C. rodentium* (Fig. S4A). Expression of *ler* (encoding the major regulator of all LEE genes) was decreased by indole (infection with *C. rodentium +tnaABC*) and serotonin (Prozac administration) but not when these two signals were elevated together (Fig. S4B).

### Indole has a reverse effect on *C. rodentium* pathogenesis in SERT knockout mice

**Fig 6:**
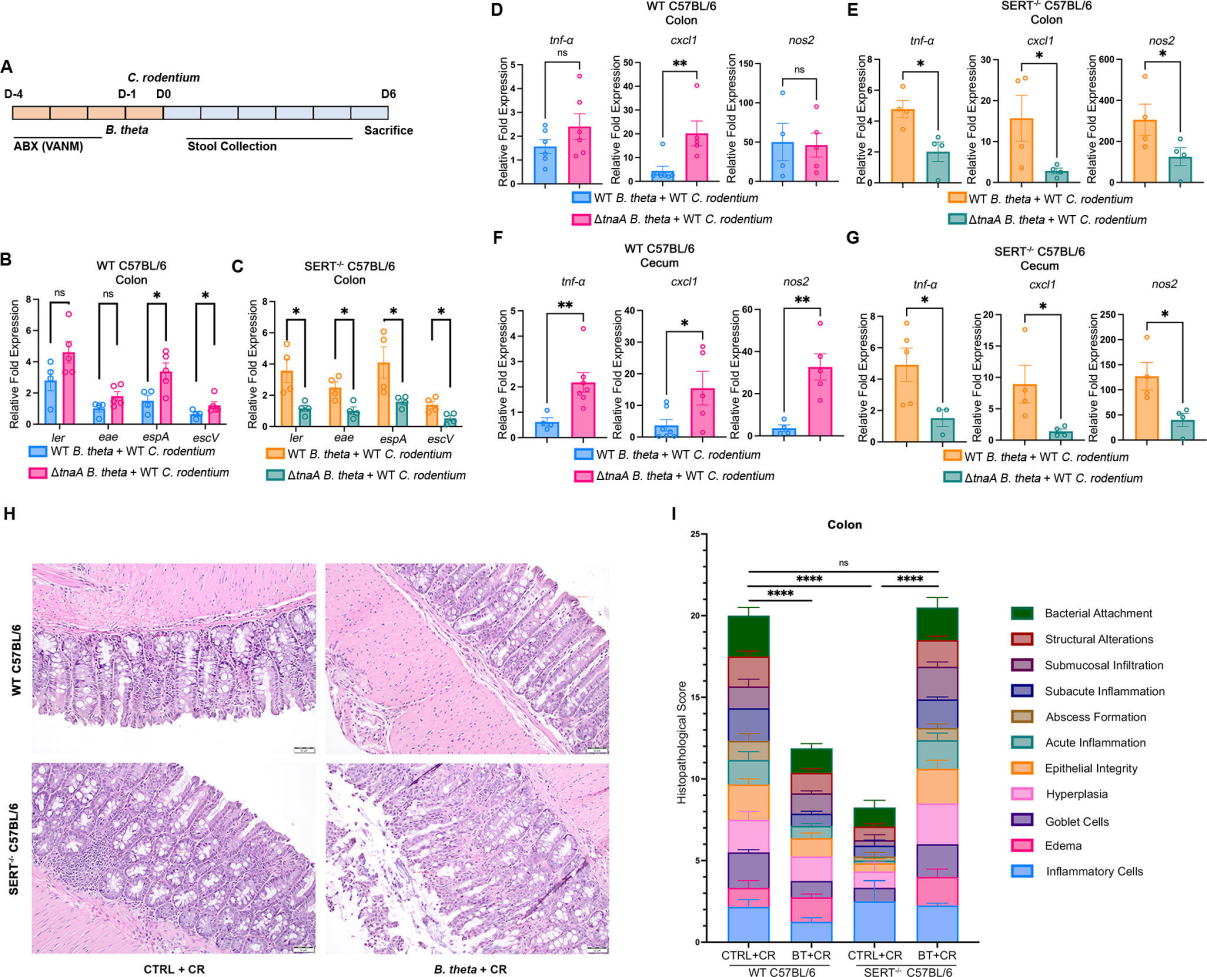
Reconstitution of microbiota to decrease indole levels decreases the virulence gene expression in the SERT^−/−^ C57BL/6 mouse strain. (**A**) Schematic of *C. rodentium* murine infection experiments with WT and SERT^−/−^ C57BL/6 mice. D, day. Prior to infection, animals were treated with a cocktail of vancomycin, ampicillin, neomycin, and metronidazole for three consecutive days. One day prior to infection, animals were inoculated with either WT *B. thetaiotaomicron* or Δ*tnaA B. thetaiotaomicron*, and the animals were infected with WT *C. rodentium*. (**B and C**) qRT-PCR analysis comparing expression of virulence genes encoded in the LEE pathogenicity island in the RNA extracted from colon tissue of (**B**) WT C57BL/6 mice and (**C**) SERT^−/−^ C57BL/6 mice colonized with *B. thetaiotaomicron* infected with *C. rodentium*, sacrificed at day 6 post-infection. *n* = 4 or 5 mice per group. Error bars represent standard errors of the mean (SEM). *P*-values were calculated using two-way ANOVA followed by Bonferroni’s multiple comparison test. *P < 0.05; ns, not significant. Fold changes were calculated relative to *rpoA* as an internal control. (D–G) qRT-PCR analysis comparing expression of inflammation markers in the extracted RNA from mice colonized with either WT *B. thetaiotaomicron* or Δ*tnaA B. thetaiotaomicron* and infected with *C. rodentium*, sacrificed at day 6 post-infection (**D**) colon tissue of WT C57BL/6 mice and (**E**) colon tissue of SERT^−/−^ C57BL/6 mice, (**F**) cecum tissue of WT C57BL/6 mice and (**G**) cecum tissue of SERT^−/−^ C57BL/6 mice. *n* = 4, 5, or 6 mice per group. Error bars represent standard errors of the mean (SEM). *P*-value is calculated using unpaired *t* test. *P < 0.05; **P < 0.01; ns, not significant. Fold changes were calculated relative to *gapdh* as an internal control. (**H**) Colon tissue samples collected from WT C57BL/6 mice and SERT^−/−^ C57BL/6 mice either colonized with WT *B. thetaiotaomicron* or not colonized and infected with WT *C. rodentium* sacrificed at day 6 post-infection, stained with hematoxylin and eosin. Scale bars, 50 µm. (**I**) Blinded histopathology scores of WT C57BL/6 mice and SERT^−/−^ C57BL/6 mice either colonized with WT *B. thetaiotaomicron* or not colonized and infected with WT *C. rodentium* and infected with WT *C. rodentium*. PBS mock infected groups were scored zero. *n* = 3 or 4 mice per group. *P*-value is calculated using two-way ANOVA followed by Bonferroni’s multiple comparison test. ****P < 0.0001; ns, not significant.

Pharmacological inhibition of SERT with Prozac alongside infection with WT and indole-producing strains of *C. rodentium* is an effective model for observing the effect of serotonin on *C. rodentium* pathogenesis in C57BL/6 mice. For a more robust animal model, we used SERT knockout mice that accumulate serotonin in the gut lumen. The presence of microbiota eliminates the difference in indole levels in animals infected with WT *C. rodentium* and *C. rodentium +tnaABC* and completely depleting the microbiota interferes with serotonin biosynthesis. To find a middle ground, we recolonized WT and SERT knockout animals after microbiota depletion with either WT *B. thetaiotaomicron* or Δ*tnaA B. thetaiotaomicron* that doesn’t produce indole (Fig. 6A). Expression of LEE genes (*ler, eae, espA,* and *escV*) increased in bacteria attached to the colon tissue of WT mice recolonized with Δ*tnaA B. thetaiotaomicron* due to lack of indole production, but this phenotype was reversed in the SERT knockout mice that had elevated serotonin levels (Fig. 6B and C). Expression of inflammation markers (*tnf-a, cxcl1,* and *nos2*) in RNA extracted from colon and cecum tissues of mice recolonized with Δ*tnaA B. thetaiotaomicron* also increased in WT mice but not in SERT knockout mice (Fig. 6D through G). When we compared the pathology of colon tissue samples, WT mice recolonized with *B. thetaiotaomicron* infected with *C. rodentium* had significantly less severe pathology compared to the infected control group with no *B. thetaiotaomicron* (no indole). SERT knockout mice infected with *C. rodentium* also presented significantly less pathology compared to WT mice, but this phenotype was reversed when we recolonized SERT knockout mice with *B. thetaiotaomicron*, again suggesting that elevation of both serotonin and indole levels simultaneously leads to these signals antagonizing each other (Fig. 6H and I).

## DISCUSSION

The mammalian gut has a rich chemistry landscape derived from both the host and the microbiota influencing the biogeography of the GI tract and the gut-brain-axis. Enteric bacterial pathogens sense and respond to these chemicals in a manner that culminates in the most spatiotemporal efficient expression of their virulence traits (1). The colon contains tryptophan derivatives including the host-derived neurotransmitter serotonin (9, 29), and the microbiota-derived indole (30).

Serotonin is synthetized in the intestine by the TpH1 enzyme in enterochromaffin cells. It is then secreted into the lumen. Serotonin signaling in the intestine is terminated by its removal by SERT, which is expressed by epithelial cells. We have previously shown that SERT knockout mice, which have higher levels of luminal serotonin, are less susceptible to *C. rodentium* infection Using the murine microbiota depleted *C. rodentium* infection model (microbiota depleted through treatment with four antibiotics), we also showed that self-produced or microbiota-derived indole decreases the expression of virulence genes, as well as *C. rodentium* virulence in mice (14). To integrate the role of serotonin and endogenous/exogenous indole signaling during murine infection, we needed to be able to address both signals in the same murine infection model. However, the initial studies with serotonin employed C57BL/6J mice, which is the strain background where the SERT knockout was constructed. The studies with *C. rodentium +tnaABC,* which was engineered to produce indole, were performed with C3H/HeJ mice that are more susceptible to disease.

Here, we integrated these models to address concomitant signaling by modulating the intestinal levels of both indole and serotonin. Separately, both signals decrease EHEC and *C. rodentium* virulence both *in vitro* and during murine infection (*C. rodentium*). However, when both signals are elevated at the same time, they are antagonistic and nullify each other’s individual effects. These data highlight the complexity of virulence regulation in response to chemical signals in the gut. In homeostasis, the levels of indole are higher in the lumen than at the epithelial lining (14). The levels of serotonin are also kept in check by its reuptake through SERT (9). It is also noteworthy that the levels of these signals also change in different intestinal compartments, with indole being more prominent in the lumen in large intestine where the microbiota is dense and its predominant membership is comprised of commensal *E. coli* and *B. thetaiotaomicron* that produce high levels of indole, compared to the small intestine (13, 14). Moreover, serotonin levels are also variable in different GI compartments being higher in the lumen of the small intestine than in the colon (9). EHEC and *C. rodentium* colonize the colon, and monitoring the levels of these tryptophan derivatives throughout the GI tract is probably important to reach the right niche, as well as achieve optimal expression of the energy expensive T3SS apparatus employed by these pathogens.

Manipulation of the levels of both signals by elevating their concentrations simultaneously leads to an antagonizing phenotype in bacterial pathogenesis. This is relevant given that serotonin luminal levels can be elevated by pharmacological interventions such as Prozac, which is widely used as an anti-depressant. Many anti-depressants and sleep aid drugs act by changing the levels of several tryptophan derivatives, such as serotonin (9). It is also notable that antibiotic usage will change the composition of the microbiota, altering the levels of indoles in the intestine. A deeper understanding of the interplay and intersection of these signaling systems is important to understand differential susceptibility to enteric pathogens. This knowledge is especially compelling given that various serotonin agonists and antagonists have been developed to treat diarrhea-predominant inflammatory bowel disease (IBS) and/or constipation (9). Moreover, different indole producing members of the microbiota differentially impact serotonergic-driven mammalian behaviors such as anxiety and depression, as well as development of drug addiction (31).

## MATERIALS AND METHODS

### Strains, plasmids, growth, and culture conditions

Strains used in this study are listed in Table S1 in the supplemental material. WT enterohemorrhagic *E. coli* (EHEC) O157:H7 strain 86-24, *Citrobacter rodentium* strain DBS770, and their isogenic mutants were routinely grown in Luria-Bertani (LB) media overnight at 37°C. *Bacteroides thetaiotaomicron* (*B. theta*) VPI-5482 and its isogenic mutants were routinely grown anaerobically overnight at 37°C in TYG medium. Aerophilic growth was performed under shaking conditions at 37°C and 200 rpm. For microaerophilic growth, cultures were left standing in a 37°C incubator. For anaerobic growth, cultures were grown in either a GasPak EZ anaerobe container system (Becton Dickinson) or in an anaerobic chamber (Coy Laboratory Products). Bacterial cultures were grown in low-glucose (1 g/L) Dulbecco’s modified Eagle medium (DMEM) until late logarithmic growth phase and harvested. Low-glucose DMEM was used to express the type III secretion system (T3SS), as these conditions were shown to induce the T3SS (32). HeLa cells were routinely cultured in high-glucose (4.5 g/L) DMEM, 10% FBS, and penicillin, streptomycin, and glutamine (PSG) cocktail.

### RNA extraction and qRT-PCR

EHEC and *C. rodentium* strains were grown in DMEM in the presence or absence of serotonin and indole until late log phase in at least three biological replicates. Harvested cells were stored in TRIzol, and RNA was extracted using the TRIzol chloroform extraction method. Extracted RNA was treated with the InvitroGen Turbo DNA-free kit and purified by SPRi (RNAClean XP). qRT-PCR was performed upon generation of cDNA libraries from 2 µg of diluted RNA using SuperScript IV reverse transcriptase, dNTPs, random primers, and DTT. cDNA was mixed with primers (Table S2) and SYBR Green, and qRT-PCR was run in QuantStudio 6 Flex (Applied Biosystems). Data were collected using QuantStudio real-time PCR software v1.3, normalized to endogenous control *rpoA* levels, and analyzed using the comparative critical threshold (C_T_) method. For all experiments, error bars indicate standard deviation (SD). A *P-value* of less than 0.05 was considered significant.

### Western blot for secreted proteins and whole cell lysates

Cultures grown in low-glucose DMEM were spun down by centrifugation at max speed for 10 min at 4°C. Supernatants were filtered using a 0.22 µm filter tube and concentrated using Amicon Ultra Centrifugal Filter with a 10 kDa molecular weight cutoff. Ten microgram of bovine serum albumin (BSA) was added to secreted protein samples as a loading control. Whole cell lysates were prepared by sonication at 70% amplitude for 15 s on and 15 s off cycle for 1.5 min, followed by centrifugation at 10,000 rpm at 4°C for 5 min to get rid of the cell debris. Protease inhibitor cocktail (PIC) was added to protect proteins from degradation. Secreted protein samples and whole cell lysates were separated on a 5%–20% SDS-polyacrylamide gel, transferred to a polyvinylidene fluoride (PVDF) membrane using Bio-Rad Trans-Blot Turbo PVDF transfer packs and blocked with 5% milk in PBS containing 0.05% Tween (PBST). Membranes were probed with either anti-EspB or anti-EspA primary antibody, washed, and incubated with a secondary rabbit antibody conjugated to streptavidin-horseradish peroxidase. Enhanced chemiluminescence (ECL) reagent was added, and the membranes were developed using the ChemiDoc touch imaging system.

### Fluorescein actin staining assays

Fluorescein actin staining (FAS) assays were performed as previously described (33). HeLa cells were grown overnight to around 80% confluency at 37°C and 5% CO_2_ on coverslips in wells containing high-glucose (4.5 g/L) DMEM. Overnight media were replaced with fresh DMEM without antibiotics prior to infection. Overnight cultures of EHEC harboring mCherry were aerobically grown in LB broth at 37°C, diluted 1:100 in low-glucose DMEM, and used to infect the HeLa cells at 37°C and 5% CO_2_. After 3 h of infection, coverslips in wells were gently washed with pre-warmed PBS and incubated in fresh low-glucose DMEM. After a total of 6 h infection, coverslips were washed, fixed, and permeabilized. The samples were treated with (FITC)-labeled phalloidin (Sigma, P5382) to visualize actin accumulation. The coverslips were mounted on slides with ProLong Diamond antifade with DAPI (Invitrogen, P36962), and ECHO Revolve R4 fluorescence microscope (Discover Echo Inc.) was used to visualize the cells. The number of HeLa cells with pedestal formation by EHEC was quantified. Replicate coverslips were quantified, and statistical analysis was performed. A *P*-value of less than 0.05 was considered significant.

### RNA sequencing library preparation and analysis

Briefly, RNAs extracted as previously described from at least four biological replicates were used for the RNA sequencing. Sequencing was run by SEQcenter. RNA libraries were prepared using Illumina’s Stranded Total RNA Prep Ligation with Ribo-Zero Plus kit and 10 bp unique dual indices (UDI). Sequencing was done on a NovaSeq X Plus, producing paired-end 150 bp reads. Demultiplexing, quality control, and adapter trimming were performed with bcl-convert (v4.1.5). Reads were mapped to the *C. rodentium* ICC168 genome (National Center for Biotechnology Information NC_013716). The data analysis was conducted using the software iDEP 0.96. Gene expression was considered significant for genes with fold change >2 and FDR < 0.05. (RNA-seq data have been deposited at the European Nucleotide Archive, GEO accession no. GSE296573).

### Indole colorimetric test

Fifty microliters of either *p*-Dimethylaminocinnamaldehyde (DMACA) indole reagent droppers or BBL indole reagent droppers (Becton Dickinson) were added onto 50 µL of bacterial cultures. The change in color of the DMACA indole reagent to green and the change in color of the BBL indole reagent to pink indicated the presence of indole.

### Murine infections

Female C57BL6/6J mice were purchased from The Jackson Laboratory at 7–8 weeks of age and housed in a specific pathogen-free facility at the University of Wisconsin—Madison. Mice in all groups were age-matched before the experiment, and all experiments were performed under IACUC-approved protocols.

### Microbiota depletion method

Prior to infection, C57BL/6 mice were orally administered a combination of vancomycin, ampicillin, neomycin, and metronidazole through oral gavage for 3 days to deplete gut microbiota. In addition to antibiotic treatment, mice were given 1 mg/mL chloramphenicol succinate sodium salt in drinking water flavored with 0.78 g/L grape-flavored Kool-Aid drink mix (Kraft Foods Global, Inc.) and 1% sucrose throughout the experiment as previously described (34). To confirm depletion of microbiota, fecal pellets were collected before and after antibiotic treatment. Pellets were resuspended in PBS and plated on brain heart infusion (BHI)-blood agar plates with no antibiotics. Colony quantifications were performed after 48 h of incubation at 37°C under aerobic and anaerobic conditions.

### Infection with *C. rodentium* with the *tna* operon

Following microbiota depletion, mice were infected with 1 × 10^9^ CFU of either WT *C. rodentium* or *C. rodentium +tnaABC* that has the *tna* operon alongside chloramphenicol administration through drinking water. Feces were collected for colony enumeration and qRT-PCR on indicated days, and survival analysis was performed. The WT *C. rodentium* strain used in this study was engineered to produce Shiga toxin (Stx) (DBS770). The experiments were performed multiple times with a total of more than 15 mice per group.

### Colony enumeration

The feces collected from infected mice were placed in microcentrifuge tubes. The feces were resuspended in PBS, normalized to feces weight, and plated on LB plates with chloramphenicol. The colonies were counted, and the groups were compared statistically by an unpaired Mann-Whitney *U* test.

### *B. thetaiotaomicron* reconstitution experiment

Following microbiota depletion, mice were infected with 3 × 10^9^ CFU of either WT *B. theta* or Δ*tnaA B. theta* via oral gavage. The next day, mice were either mock infected with PBS or infected with 1 × 10^9^ CFU of WT *C. rodentium*. Feces were collected for colony enumeration and qRT-PCR on indicated days. The experiments were performed multiple times with a total of more than 15 mice per group.

### Tissue and content collection for RNA isolation and qRT-PCR

Mice were euthanized at the indicated time points, and tissue and content from the colon and cecum were collected. The tissue was washed in PBS three times to remove any residual fecal content. The content and tissues were collected in tubes, and the tubes were snap-frozen in liquid nitrogen and stored in −80°C until use. RNA was isolated from content and tissue from each individual mouse, and qRT-PCR was performed as described earlier using QuantStudio 6 Flex (Applied Biosystems).

### Prozac experiments

Seven to eight weeks old female C57BL/6J mice were purchased from The Jackson Laboratory. Starting 2 days prior to infection, one group of mice was administered fluoxetine (Prozac) at 20 mg/kg per mouse per day by oral gavage, and the other group was given an equivalent amount of water by oral gavage. The microbiota of the mice was depleted, and they were infected with 1 × 10^9^ CFU of either WT *C. rodentium* or *C. rodentium +tnaABC* strain. The mice in all the groups were age-matched before the experiment.

### Gnotobiotic animal experiments

Gnotobiotic C57BL6/J mice colonies were established in the University of Wisconsin—Madison BRMS Mouse Breeding and Research Services Core. Gnotobiotic animals at 7–8 weeks of age were transferred into three separate isolators for experimentation. Animals from each isolator were either mock-infected with PBS or infected with 1 × 10^9^ CFU of WT *C. rodentium* or *C. rodentium +tnaABC*. In each isolator, starting 2 days prior to infection, half of the animals received 20 mg/kg Prozac per mouse per day by oral gavage or PBS for vehicle.

### SERT knockout experiments

A colony of B6.129 (Cg)-Slc6a4^tm1Kpl^/J mice that are homozygous for the serotonin transporter targeted mutation was established in the University of Wisconsin—Madison BRMS Mouse Breeding and Research Services Core. The mice strain was cryorecovered, and two founder pairs of heterozygous male and female mice for Slc6a4^tm1Kpl^ were purchased from The Jackson Laboratory (JAX stock #008355, Bar Harbor, ME). To determine the genotype of the pups, PCR analysis was performed as described previously. High anxiety is observed in homozygous and heterozygous females and can lead to undesirable behaviors maternally, which makes it hard to obtain substantial numbers. Experiments were performed multiple times whenever litter size was large enough. Seven to eight weeks old homozygous male and female mice and WT control littermates were transferred to a specific pathogen-free facility. WT and SERT knockout mice were either recolonized with WT *B. thetaiotaomicron* or ΔtnaA *B. thetaiotaomicron* and infected with WT *C. rodentium* or not. Some control groups were given PBS instead of recolonization with *B. thetaiotaomicron,* and some control groups were given PBS instead of infection with *C. rodentium*.

### Histopathological staining and analysis

Portions of the distal colon and cecum were harvested 6 days post-infection when the pathology caused by *C. rodentium* infection is high. The tissues were washed in PBS and preserved in 10% formalin solution (Sigma). Tissues were fixed, embedded in paraffin, sectioned, and stained with hematoxylin and eosin in the University of Wisconsin—Madison Comparative Pathology Laboratory. The histological score was obtained based on the bacterial attachments, structural alterations, submucosal infiltration, subacute inflammation, acute inflammation, abscess formation, epithelial integrity, hyperplasia, number of goblet cells, edema formation, and number of inflammatory cells. The histological analysis was performed blindly by a single examiner.

### Quantification and statistical analysis

For all *in vitro* experiments, a Student’s unpaired *t*-test for comparing two groups and a one-way ANOVA for comparing more than two groups were used to determine the statistical significance. The Bonferroni-Dunn method was used for multiple comparison correction when required. For all *in vivo* experiments, statistical significance was determined using the nonparametric Mann-Whitney *U* test. The log rank (Mantel-Cox) test was used to calculate statistics for survival analysis. Details of the numbers of biological samples and animals used can be found in the figure legends.

## SUPPLEMENTAL MATERIAL

Figure S1 mbio.02067-25-s0001.pdf Serotonin and indole have no effect on EHEC growth.

Figure S2 mbio.02067-25-s0002.pdf Additional genes differentially regulated by the presence of serotonin and indole.

Figure S3 mbio.02067-25-s0003.pdf Serotonin and indole individually decrease *C. rodentium* virulence gene expression but antagonize each other.

Figure S4 mbio.02067-25-s0004.pdf Manipulation of serotonin levels with Prozac in gnotobiotic mice prevents the decrease in *C. rodentium* pathogenicity caused by indole production.

legends mbio.02067-25-s0005.docx Supplemental figure legends.

Table S1 mbio.02067-25-s0006.docx Strains.

Table S2 mbio.02067-25-s0007.docx Primers.

ASM does not own the copyrights to Supplemental Material that may be linked to, or accessed through, an article. The authors have granted ASM a non-exclusive, world-wide license to publish the Supplemental Material files. Please contact the corresponding author directly for reuse.
