# Supplementary figures and images for: Combinatorial effects of tryptophan derivatives serotonin and indole on virulence modulation of enteric pathogens
Source: mBio. 2025 Aug 25;16(10):e02067-25. doi: 10.1128/mbio.02067-25 (PMC12506081; doi:10.1128/mbio.02067-25)

**A**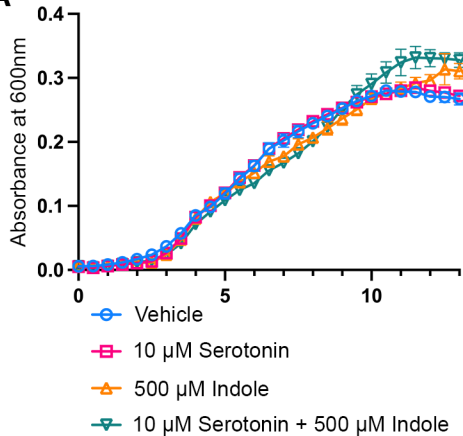**B**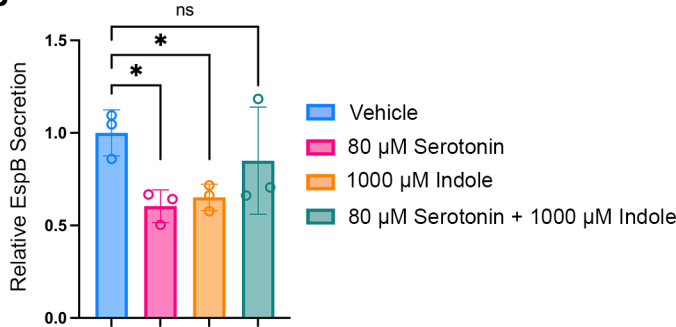

Supplement: Figure S1 — Serotonin and indole have no effect on EHEC growth. [file mbio.02067-25-s0001.pdf]

**A**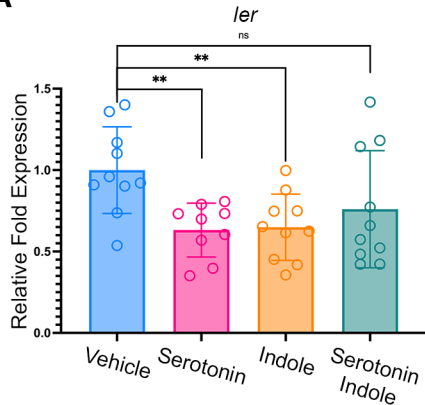**B**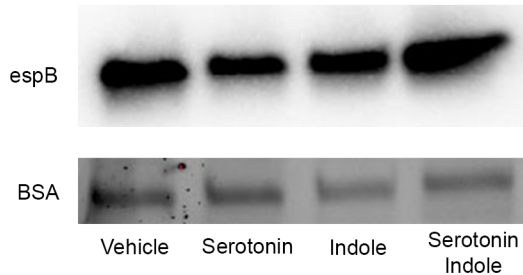

Supplement: Figure S3 — Serotonin and indole individually decrease C. rodentium virulence gene expression but antagonize each other. [file mbio.02067-25-s0003.pdf]
