# Supplementary material for: Combinatorial effects of tryptophan derivatives serotonin and indole on virulence modulation of enteric pathogens
Source: mBio. 2025 Aug 25;16(10):e02067-25. doi: 10.1128/mbio.02067-25 (PMC12506081; doi:10.1128/mbio.02067-25)

	Gene	Serotonin	Indole	Serotonin + Indole
Quorum Sensing 2CS	qseB	-0.38	-0.50	-0.28
	qseC	-1.02	-0.71	-0.30
Shiga toxin	stx2A	-0.67	-1.06	-0.34
	stx2B	-0.90	-0.90	-0.33

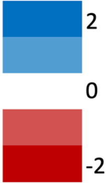

Supplement: Figure S2 — Additional genes differentially regulated by the presence of serotonin and indole. [file mbio.02067-25-s0002.pdf]
