# Supplementary material for: Combinatorial effects of tryptophan derivatives serotonin and indole on virulence modulation of enteric pathogens
Source: mBio. 2025 Aug 25;16(10):e02067-25. doi: 10.1128/mbio.02067-25 (PMC12506081; doi:10.1128/mbio.02067-25)
Supplement: Figure S4 — Manipulation of serotonin levels with Prozac in gnotobiotic mice prevents the decrease in C. rodentium pathogenicity caused by indole production. [file mbio.02067-25-s0004.pdf]

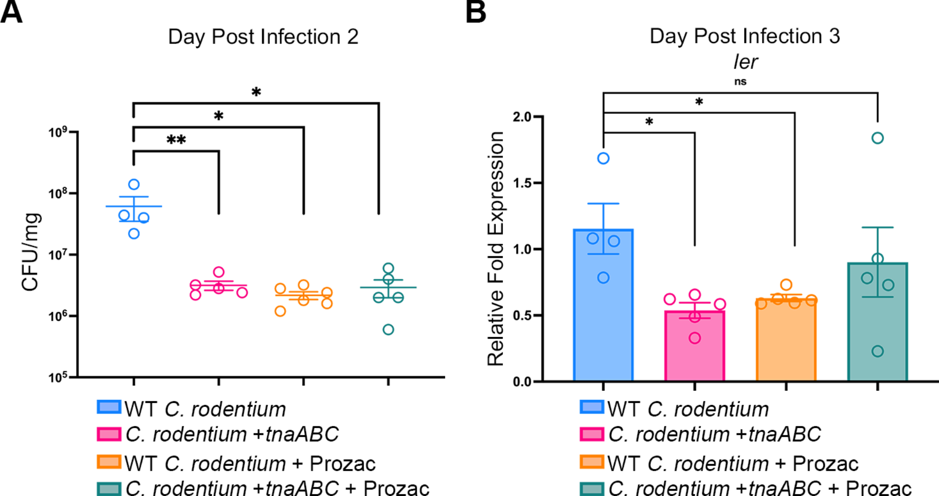

A
Day Post Infection 2
CFU/mg
10^9
10^8
10^7
10^6
10^5
**
*
*
B
Day Post Infection 3
ler
ns
*
*
Relative Fold Expression
2.0
1.5
1.0
0.5
0.0
WT *C. rodentium*
C. rodentium +tnaABC
WT *C. rodentium* + Prozac
C. rodentium +tnaABC + Prozac
