# Supplementary material for: Combinatorial effects of tryptophan derivatives serotonin and indole on virulence modulation of enteric pathogens
Source: mBio. 2025 Aug 25;16(10):e02067-25. doi: 10.1128/mbio.02067-25 (PMC12506081; doi:10.1128/mbio.02067-25)
Supplement: legends — Supplemental figure legends. [file mbio.02067-25-s0005.docx]

**FIG S1** Serotonin and indole have no effect on EHEC growth. (A) Growth curves of WT EHEC cells grown with or without 10 µM serotonin and 500 µM indole, and absorbances of growth cultures were measured at an OD_600_ every 30 minutes. Error bars represent standard deviation (SD). (B) ELISA for EspB secretion of WT EHEC cells grown with or without serotonin and indole for 6 hours, n= 3. Error bars represent standard deviation (SD). *P*-value was calculated using unpaired *t* test. *, *p*<0.05, *ns*, not significant.

**FIG S2** Additional genes differentially regulated by the presence of serotonin and indole. Heatmap showing differential regulation of genes encoding for the QseBC two-component system and Shiga toxin in EHEC treated with 10 µM serotonin, 500 µM indole, or both.

**FIG S3** Serotonin and indole individually decrease *C. rodentium* virulence gene expression, but antagonize each other. (A) qRT-PCR analysis comparing expression of virulence gene *ler* in *C. rodentium* treated with vehicle, 10 µM serotonin for 2 hours, 500 µM indole for 4 hours, or both. Error bars represent standard deviations (SD). *P*-value is calculated using unpaired *t* test. *, *p*<0.05; **, *p*<0.01; ***, *p*<0.001; ns, not significant. Fold changes were calculated relative to *rpoA* as an internal control. Data are representative of multiple experiments with at least three biological replicates. (B) Western blot on EspB protein from the secreted protein samples concentrated with an Amicon Ultra Centrifugal Filter with 10 kDa molecular weight cutoff from supernatant of WT *C. rodentium* treated with vehicle, 10 µM serotonin for 2 hours, 500 µM indole for 4 hours, or both. 10 µM BSA was added as a loading control.

**FIG S4** Manipulation of serotonin levels with Prozac in gnotobiotic mice prevents the decrease in *C. rodentium* pathogenicity caused by indole production. Gnotobiotic C57BL/6 mice were infected with either WT *C. rodentium* or *C. rodentium* +*tnaABC* strain that harbors the *tna* operon to produce indole. Starting two days prior to infection, mice were administered 20 mg/kg Prozac or PBS for vehicle through oral gavage throughout the experiment. *n*=4, 5, or 6 mice per group (A) Enumeration of CFUs of *C. rodentium* loads recovered from stools on day 2 post-infection. Error bars represent standard errors of the mean (SEM). Groups were compared using unpaired *t*-test. *, *p*<0.05; **, *p*<0.01. (B) qRT-PCR analysis comparing expression of *ler* in the RNA extracted from stool collected at day 3 post-infection. Error bars represent standard errors of the mean (SEM). *P*-value is calculated using unpaired *t* test. *, *p*<0.05; ns, not significant. Fold changes were calculated relative to *rpoA* as an internal control.

**Table S1** Strains used in the study.

**Table S2** Primers used in the study.
