# Supplementary material for: Combinatorial effects of tryptophan derivatives serotonin and indole on virulence modulation of enteric pathogens
Source: mBio. 2025 Aug 25;16(10):e02067-25. doi: 10.1128/mbio.02067-25 (PMC12506081; doi:10.1128/mbio.02067-25)
Supplement: Table S1 — Strains. [file mbio.02067-25-s0006.docx]

**Table S1** Strains used in the study.

| **Strains** | **Description** | **Reference** |
| --- | --- | --- |
| EHEC WT | *E. coli* O157:H7 86-24 Clinical isolate | (Griffin et al., 1988) |
| DBS770 | WT C. rodentium | (Mallick et al., 2012) |
| DBS770 +tnaABC | C. rodentium with tna operon | Kumar et al. 2019 |
| *B. thetaiotaomicron* VPI-5482 | WT *B. thetaiotaomicron* | ATCC 29148 |
| *B. thetaiotaomicron* Δ*tnaA* | *Isogenic tnaA* deletion mutant in *B. thetaiotaomicron* | Kumar et al. 2019 |
