# Supplementary material for: Combinatorial effects of tryptophan derivatives serotonin and indole on virulence modulation of enteric pathogens
Source: mBio. 2025 Aug 25;16(10):e02067-25. doi: 10.1128/mbio.02067-25 (PMC12506081; doi:10.1128/mbio.02067-25)
Supplement: Table S2 — Primers. [file mbio.02067-25-s0007.docx]

**Table** S2 Primers used in the study.

| **Primer** | **Sequence 5’ to 3’** |
| --- | --- |
|  | **qRT-PCR for EHEC** |
| *rpoA* F | GTGACCCTTGAGCCTTTAGAG |
| *rpoA* R | ACACCATCAATCTCAACCTCG |
| *espA* F | AGCTATTTGAGGAACTCGGTG |
| *espA* R | CATCTTTTGTGCCGTGGTTG |
| *eae* F | TGGGATGTTCAACGGTAAGTC |
| *eae* R | TTTAACCTCAGCCCCATCAC |
|  | **qRT-PCR for *C. rodentium*** |
| CR *rpoA* F | ACGTCAGCCGGAAGTGAAAGAAGA |
| CR *rpoA* R | AGCGGACAGTCAATTCCAGATCGT |
| CR *ler* F | ACAGTTTGAATCTCCTGCTCACGC |
| CR *ler* R | ATTTCGCCCACAACAAGCCCATAC |
| CR *espA* F | ACGAGGTAACAACCATGCGAGTGT |
| CR *espA* R | CTGCCTGGCATTGCTTTCCAGAAT |
| CR *tir* F | ATCAGATATCTCGCAAGCTCG |
| CR *tir* R | CAACTCCATCTCCCATTCCTG |
| CR *eae* F | TGCGAAAGATACAGCCCTTAG |
| CR *eae* R | ACCTCTGCCGTTCCATAATG |
| CR *escV* F | GGGCGATGAAGTTTGTAAAAGG |
| CR *escV* R | CCAACCGACAATACAGAAAACAG |
|  | **qRT-PCR for Inflammation Markers** |
| *gapdh* F | GGTGAAGGTCGGAGTCAACGGA |
| *gapdh* R | GAGGGATCTCGCTCCTGGAAGA |
| *tnfa* F | CATCTTCTCAAAATTCGAGTGACAA |
| *tnfa* R | CCAGCTGCTCCTCCACTTG |
| *cxcl1* F | ACTGCACCCAAACCGAAGTC |
| *cxcl1* R | TGGGGACACCTTTTAGCATCTT |
| *nos2* F | CCTCCTTCAGGTCACTITGGTAGG |
| *nos2* R | TTGGGTCTTGTTCAGCCACGG |
|  | **PCR for SERT Knockout Mice** |
| 30801 Wild type Forward | CCT GTC TGT CAT TGG CTA TGC |
| 30802 Common | GCT GAC TGG AGA TCA GGC TA |
| oIMR6773 Mutant Forward | TGG CTA CCC GTG ATA TTG CT |
